# Promiscuous attraction of ligands within the ATP binding site of RyR2 promotes diverse gating behaviour

**DOI:** 10.1038/s41598-018-33328-8

**Published:** 2018-10-09

**Authors:** Chris Lindsay, Mano Sitsapesan, Wei Mun Chan, Elisa Venturi, William Welch, Maria Musgaard, Rebecca Sitsapesan

**Affiliations:** 1 Department of Pharmacology, University of Oxford, Oxford, UK; 2 Department of Chemistry, Chemistry Research Laboratory, University of Oxford, Oxford, UK; 3 University of Nevada School of Medicine, Department of Biochemistry, Reno, Nevada USA; 4 Structural Bioinformatics and Computational Biochemistry, Department of Biochemistry, University of Oxford, Oxford, UK; 5 Present Address: Department of Chemistry and Biomolecular Sciences, University of Ottawa, Ottawa, Canada

## Abstract

ATP is an essential constitutive regulator of cardiac ryanodine receptors (RyR2), enabling small changes in cytosolic Ca^2+^ to trigger large changes in channel activity. With recent landmark determinations of the full structures of RyR1 (skeletal isoform) and RyR2 using cryo-EM, and identification of the RyR1 ATP binding site, we have taken the opportunity to model the binding of fragments of ATP into RyR2 in order to investigate how the structure of the ATP site dictates the functional responses of ligands attracted there. RyR2 channel gating was assessed under voltage-clamp conditions and by [^3^H]ryanodine binding studies. We show that even the triphosphate (PPPi) moiety alone was capable of activating RyR2 but produced two distinct effects (activation or irreversible inactivation) that we suggest correspond to two preferred binding locations within the ATP site. Combinations of complementary fragments of ATP (Pi + ADP or PPi + AMP) could not reproduce the effects of ATP, however, the presence of adenosine prevented the inactivating PPPi effects, allowing activation similar to that of ATP. RyR2 appears to accommodate diverse types of molecules, including PPPi, deep within the ATP binding site. The most effective ligands, however, have at least three phosphate groups that are guided into place by a nucleoside.

## Introduction

Ryanodine receptors (RyRs) are the principal ion channels responsible for controlling the sarcoplasmic reticulum (SR) Ca^2+^ release that is required for cardiac cell contraction. Of the three mammalian RyR isoforms, RyR2 is found predominately in cardiac tissue, and its opening is prompted by Ca^2+^ entering the cell through L-type Ca^2+^-channels^1^. There are many physiological regulators of RyR channels but ATP is one of the most important because it is a constitutive and highly efficacious activator, acting synergistically to potentiate the effects of cytosolic Ca^2+ ^^2–5^. ATP is present at millimolar levels in cardiac cells and, in the presence of activating levels of cytosolic Ca^2+^, enables RyR2 to open to high Po levels^6^. The crucial role of ATP in Ca^2+^ release has been demonstrated by the finding that Ca^2+^-induced-Ca^2+^-release in cardiac myocytes is abolished following severe reductions in ATP concentrations^5^. Prolonged periods of myocardial ischemia lead to reduced intracellular levels of ATP and increases the levels of breakdown products of ATP, including agents such as inorganic phosphate (Pi), adenosine and AMP^7^. We and others have demonstrated that many of these metabolites can modulate RyR2 channel gating, some by competing with ATP for its binding sites on RyR2^3,6,8–10^. Since the changes in RyR2 channel regulation that occur during ischaemia may contribute to the changes in intracellular [Ca^2+^] that predispose towards arrhythmias, it is important to understand how ATP and its metabolites affect RyR2 gating. The RyR2 nucleotide binding site is therefore a potential target for cardiac therapeutic agents not only in ischemia but also in other disease states where arrhythmias arise because of irregularities in SR Ca^2+^-release through RyR2.

**Figure 1:**
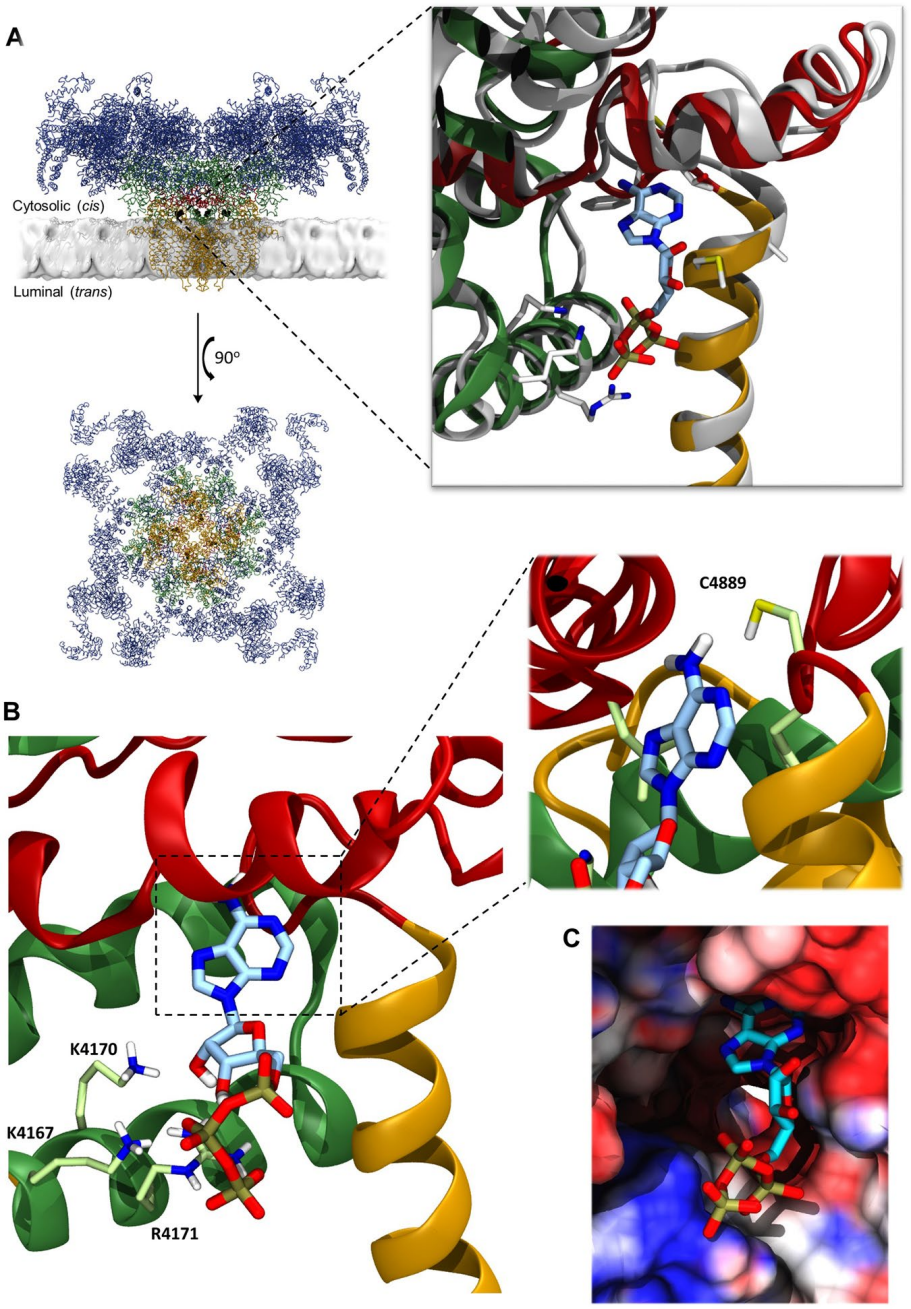
Comparison of RyR1 and RyR2 at the ATP binding site illustrating key residues for interaction with the ATP molecule. (**A**) The overall structure of RyR2 (PDB-ID 5GOA) is depicted in cartoon representation, both in the lipid bilayer and from above^16^. The membrane position was taken from the Orientations of Proteins in Membranes database^38^. Residues 1–3636 are shown in blue, 3636–4207 in green, 4480–4888 (transmembrane domain) in orange and 4889–4967 (CTD region) in dark red. The suggested ATP binding site in each chain is indicated by four black dots and the inset to the right shows a magnification of one of these sites. Each identical ATP site (inset) consists of a fairly hydrophobic pocket, and a more exposed region lined by positively charged lysine and arginine residues. RyR1 (PDB-ID 5TAS) is shown in grey cartoon representation, with important residues indicated and with ATP in the binding site. RyR2, coloured as above, is overlaid for comparison to illustrate the close similarity with RyR1 at this site. (**B**) The RyR2 ATP site is further expanded to show the Autodock-predicted pose for ATP which is most similar to the ATP binding mode observed in the RyR1 cryo-EM structure; important residues are indicated. The inset shows an enlarged view of the adenine region with the C4889 residue shown. (**C**) The RyR1 (PDB-ID 5TAS) ATP site shown in surface representation with an ATP molecule bound. The surface is coloured by the electrostatic potential, going from dark blue (most positive), through white (neutral) to red (negative).

While single-channel function of RyR2 has been studied for some time, the large size of this protein has hampered attempts to determine a full high-resolution crystal structure of any of the RyRs, with only some regions and domains being reported^11–15^. More recently, however, advances in cryo-electron microscopy (cryo-EM) have allowed insight into the structural features of the protein, with a full RyR2 (pig) structure being determined in 2016 to an overall resolution of approximately 4.2 Å^16^. The RyR2 ATP site has not yet been identified, however, des Georges *et al*. (2016) recently reported a putative ATP binding site in the skeletal isoform (rabbit) of RyR (RyR1)^17^, in a structure determined to a resolution of 6.2 Å. The ATP site is located between residues 4211 and 4985 (RyR1 numbering), with four identical sites per RyR tetramer. The site consists of a fairly hydrophobic cavity and a more exposed region lined by several positively charged residues (Fig. 1). Of the 50 residues which have at least one atom within 10 Å of ATP, only three differ between RyR1 (rabbit) and RyR2 (pig), namely RyR1 M4207 (K4163 in RyR2), RyR1 S4965 (N4896 in RyR2) and RyR1 E4981 (Q4912 in RyR2). None of these three residues form direct contacts to the bound ATP molecule, and their side chains point away from the binding site. Hence, the suggested ATP site is almost completely conserved between the RyR1 and RyR2 isoforms. In the structures (PDB-IDs 5TAS and 5GOA), the root-mean-square deviation (RMSD) between the C_α_ atoms of these 50 residues in a single ATP site is 1.84 Å, confirming that the site is structurally conserved as well.

**Figure 2:**
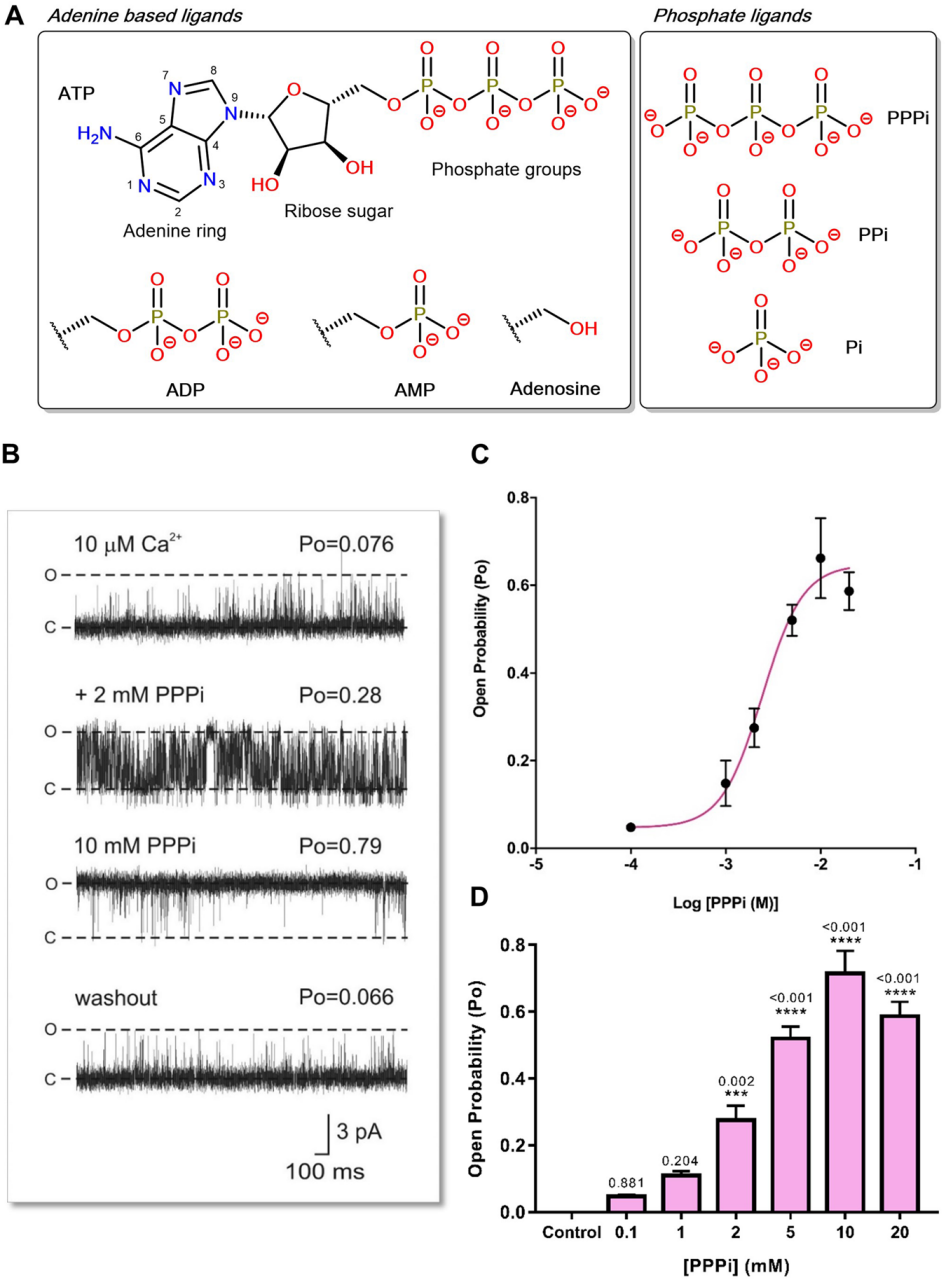
Reversible effects of PPPi on RyR2 channel gating. (**A**) A molecule of adenosine triphosphate (ATP) is shown with the adenine core, ribose ring and phosphate groups. The adenine-based ligands and phosphate ligands investigated in this study are illustrated, and their comparison to ATP are indicated. (**B**) Representative current fluctuations through a single RyR2 channel incorporated into a bilayer are shown. In the top trace, the channel is activated by 10 μM cytosolic Ca^2+^ alone. 2 mM and 10 mM PPPi are then added sequentially to the cytosolic chamber as indicated. After perfusing away the PPPi (washout), Po returns to control levels. The holding potential was 0 mV. O and C represent the open and closed channel levels, respectively. The Po above each trace refers to the value determined over 3 min. (**C**) The relationship between PPPi concentration and RyR2 Po for channels where the effect of PPPi was completely reversible. Increasing concentrations of PPPi were added sequentially. The EC_50_ value was 2.4 mM and the Hill coefficient was 2.03. (**D**) The bar chart illustrates that the increases in Po in the presence of PPPi were highly significant at high concentrations. Mean values ± SEM are shown, and p values relative to control are indicated above each bar (n = 6; ***p < 0.001; **p < 0.01; *p < 0.05).

The availability of RyR structures now enables us to model the binding of ligands into the ATP site so that the structural features of ATP-like molecules that govern their ability to activate RyR2 can be investigated. We have previously shown that many adenine based compounds can activate RyR2 but that the triphosphate group of ATP (Fig. 2A) is the key portion of the molecule that enables ATP to cause high open probability (Po)^6,10^. To investigate the role of phosphates in stabilising the open channel, we have now examined how the phosphate groups alone can manipulate RyR2 channel gating and whether different fragments of ATP, when added together, can act like an intact ATP molecule.

Our results demonstrate that electrostatic interactions generated by the triphosphate region of the ATP molecule are the most important element for inducing long open states in RyR2 and that the triphosphate groups alone are capable of activating RyR2. The adenine base plays a crucial role, however, by encouraging the correct positioning of the phosphates in the binding site. This is essential for creating high affinity binding and for preventing channel inactivation.

## Methods

### Single-channel recordings

Heavy SR membrane vesicles were prepared from sheep cardiac muscle as previously described^18^. Sheep hearts (Suffolk breed) were obtained from an abattoir and were 8–10 months old, mixed sex. SR vesicles were frozen rapidly in liquid nitrogen and stored at −80 °C. Vesicles were fused with planar phosphatidylethanolamine lipid bilayers as previously described^18^. The vesicles fused in a fixed orientation such that the *cis* chamber corresponded to the cytosolic space and the *trans* chamber to the SR lumen. The *trans* chamber was held at ground and the *cis* chamber held at potentials relative to ground. After fusion, the *cis* chamber was perfused with 250 mM HEPES, 125 mM Tris, 10 μM free Ca^2+^ buffered with EGTA and CaCl_2_, pH 7.2. The *trans* chamber was perfused with a solution containing 250 mM glutamic acid, 10 mM HEPES, pH 7.2 with Ca(OH)_2_ (free [Ca^2+^] ~ 50 mM). The free [Ca^2+^] and pH of the solutions were measured at 22 °C using a calcium electrode (Orion 93-20, Thermo Fisher Scientific, UK) and Ross-type pH electrode (Orion 81-55, Thermo Fisher Scientific, UK) and were maintained constant as described previously in detail^18^. The cytosolic [Ca^2+^] was maintained at 10 µM after additions of PPPi, by buffering with EGTA and CaCl_2_ as previously described^19^.

### Single-channel analysis

Single-channel recordings were digitized at 20 kHz and low-pass filtered at 800 Hz. Po was determined over 3 min of continuous recording at 0 mV using 50% threshold analysis^20^ in Clampfit 10.6 (Molecular Devices, USA) as previously described^18,21^. Where Po values are shown in figures, the Po above each trace refers to the value determined over 3 min for that recording. Where more than one channel incorporated into the bilayer, Po is reported as an average (total Po divided by number of channels).

### [^3^H]ryanodine binding

Membrane vesicles were diluted to 50–100 μg protein/ml and incubated at 37 °C in 250 mM HEPES, 80 mM Tris, 10 μM free Ca^2+^, pH 7.2 with 5 nM [^3^H]ryanodine for 90 min. Following incubation, the samples were diluted with 5 ml of ice-cold buffer and filtered through Whatman GF-B filters. Filters were washed with a further 3 × 5 ml aliquots of buffer and counted in 10 ml aqueous counting scintillant the following day. Non-specific binding was determined from incubations to which 5 μM unlabelled ryanodine had been added. All incubations were performed in triplicate. Adjustments to the ionic strength of the solutions were made as previously described^19^. The free [Ca^2+^] and pH of the solutions used during the incubation period were determined at 37 °C using a calcium electrode (Orion 93-20, Thermo Fisher Scientific, UK) and Ross-type pH electrode (Orion 81-55, Thermo Fisher Scientific, UK) as described above for solutions maintained at 22 °C^18^. The free [Ca^2+^] of the incubation solutions was maintained at 10 μM in the presence or absence of various ligands by buffering with EGTA and CaCl_2_.

### Docking

Docking calculations were performed using the ATP-free, pig RyR2 protein structure (PDB accession number 5GOA, resolution 4.2 Å) determined in the open channel state^16^. All residues within 10 Å of the ATP site are completely conserved between pig^16^ (structure) and sheep (our functional data). The downloaded pdb-like files were combined into a single file. M4885 was taken as the approximate centre of the ATP site, and only residues within 50 Å of this residue in chain A were retained. Bound Zn^2+^ ions in the vicinity of the ATP site were found to have minimal effect on the docking of ATP and so were removed for the docking calculations. All ligands were prepared in Maestro 10.7 (Schrodinger, LLC, New York, 2016 academic version). Gasteiger partial charges for the ligands were generated using Antechamber in AmberTools14^22^. Protein and ligands were prepared for docking in AutoDock Tools, letting AutoDock Tools generate the Gasteiger partial charges for the protein. Docking calculations were subsequently performed with AutoDock 4.2.3^23,24^. The gridbox was 50 × 50 × 50 Å^3^centred around the central M4885 residue. Residues K4167, K4170 and R4171 were treated as flexible. All other settings were default unless otherwise stated. Initial docking calculations were unsuccessful in producing poses in which ATP bound in a similar way to the binding mode suggested in the RyR1 structure. Structural comparisons with RyR1 (PDB 5TAS) suggested that M4885 hindered access to the hydrophobic pocket. Hence, M4885 was treated as flexible in a test run, which produced some poses in which the methionine side chain had changed orientation, allowing the adenine part of ATP to bind in the hydrophobic pocket. This prompted us to adjust the conformation of the M4885 sidechain to a rotamer more like the one observed in the ATP-bound RyR1 structure using the ‘rotamer toggle’ functionality in Pymol^25^. This alternative conformation of M4885 was used for all docking results presented here. To investigate the ability to bind multiple ligands at the same time, the preferred docking poses (those poses most similar to the observed orientation of ATP in RyR1 seen by des Georges *et al*.^17^) for ADP, AMP and adenosine were incorporated into the protein PDBQT file, and docking was subsequently performed with Pi, PPi and PPPi, respectively. All settings were retained throughout both rounds of docking. Structural images were prepared in VMD^26^.

### Statistical analysis

Statistical analysis was performed using GraphPad Prism 7. Mean ± SEM are shown where the number of experiments ≥5. Where appropriate, Student’s t-test was used to assess the difference between mean values. Where comparison of three or more groups was required, we used ANOVA followed by a modified t-test^27^. A *p* value of <0.05 was taken as significant. In all cases where sheep HSR was used, data was obtained from at least 5 different membrane preparations from 5 different animals. Binding energies were calculated using Autodock using the generic algorithm and the resulting poses were clustered using AutoDockTools 1.5.6 to a tolerance of 2.0 Å^28^. We compared the docking of ligands into the RyR2 cryo-EM structure that does not have ATP bound (Peng *et al*.^16^), against the RyR1 cryo-EM structure that does have ATP bound (des Georges *et al*. 2016). For the preferred binding mode in RyR2, the Pearson correlation coefficient (R^2^) was used to assess the likely correlation between the binding energy obtained from the docking procedure and the experimentally observed activation of RyR2 caused by each ligand.

### Materials

[^3^H]ryanodine was purchased from New England Nuclear Ltd. Unlabelled ryanodine was purchased from Calbiochem (Nottingham, UK). PPPi was obtained from Sigma-Aldrich (Sigma-Aldrich Company Ltd. Gillingham, Dorset, UK) and all other chemicals were obtained from BDH (BDH Biochemical Laboratories, Poole, UK). Aqueous counting scintillant was purchased from Amersham International (Amersham, UK). Deionised water (Millipore, Harrow, UK) and all other aqueous solutions used in single-channel experiments were filtered through a membrane with a 0.45 μM diameter pore.

## Results

### Effect of PPPi on single-channel gating

The adenine-based compounds and phosphate ligands used in this study are shown in Fig. 2A. The effects of PPPi on RyR2 gating were investigated by adding PPPi to the cytosolic side of a single RyR2 channel reconstituted into a bilayer (Fig. 2B). In this example, PPPi activated the channel to a high Po and the effect was fully reversible after perfusing away the PPPi. This type of activation by PPPi was observed in 7 out of 15 experiments (47%). The relationship between [PPPi] and Po for these experiments is shown in Fig. 2C. The EC_50_ value was 2.4 mM and the Hill coefficient was 2.03 indicating that multiple PPPi molecules must bind for maximum effect. The maximum Po obtained was 0.647 ± 0.126 (SEM; n = 7) and was observed at 10 mM PPPi. This is lower than the maximum Po attainable with ATP under identical experimental conditions (approximately 0.9)^3^ but is higher than that observed with adenine nucleotides containing fewer than three phosphates or a reduced negative charge in the region of the phosphate groups as with AMP-PCP (Po approximately 0.4)^10^.

**Figure 3:**
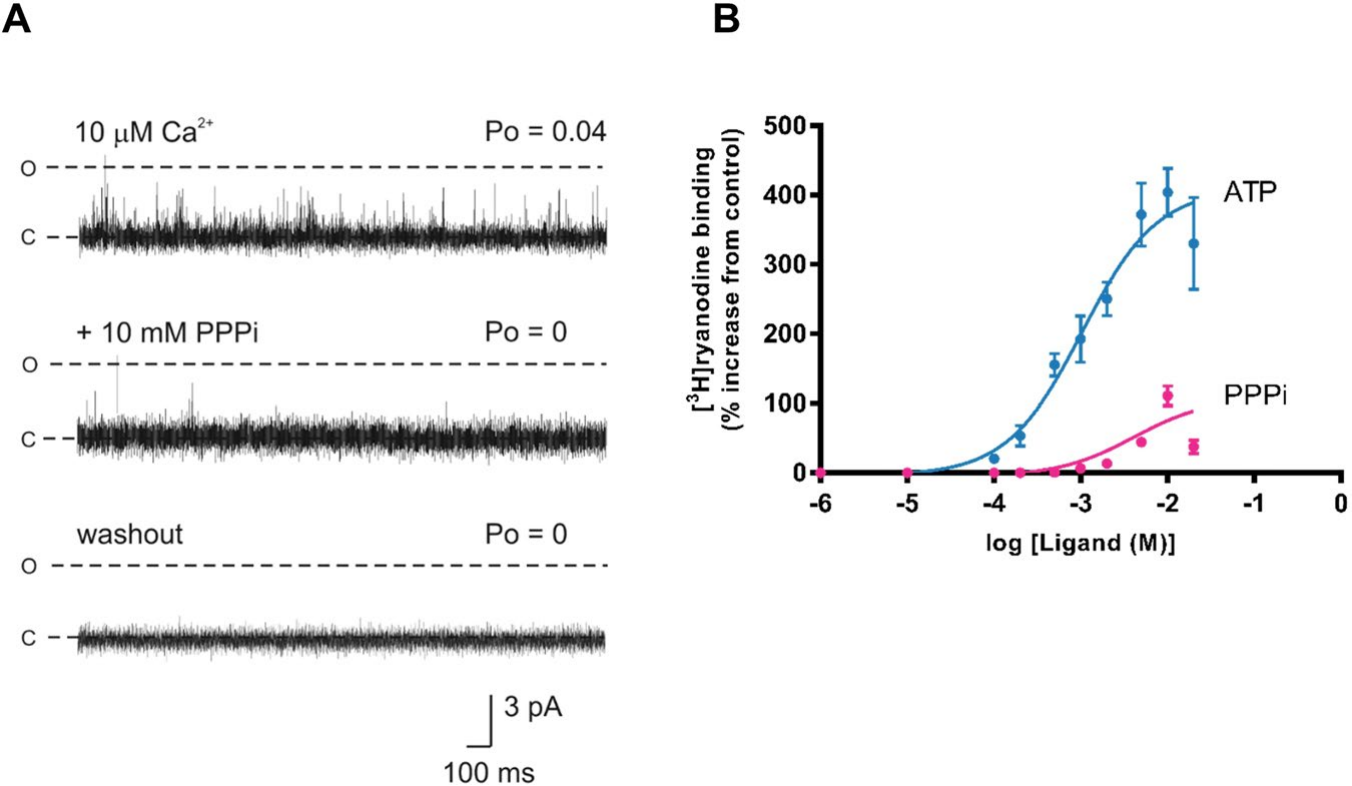
Irreversible RyR2 inactivation by PPPi. (**A**) The top trace shows a typical single RyR2 channel activated by 10 μM cytosolic Ca^2+^ alone. The subsequent trace shows an example of the inactivating effects of 10 mM PPPi. Occasional, very brief openings were observed initially as shown in the middle trace but after 140 s no further openings were detected. The bottom trace shows that after perfusing away the PPPi from the cytosolic chamber, the channel remains shut (no openings during the 3 min recording period). The holding potential was 0 mV. O and C represent the open and closed channel levels respectively. (**B**) Comparison of the stimulation of [^3^H]ryanodine binding to vesicles of sheep cardiac heavy SR by ATP (blue) and PPPi (pink). The results are expressed as a percentage of the control binding at 10 μM cytosolic Ca^2+^ which was 0.18 ± 0.02 pmol [^3^H]/mg protein (SEM; n = 10). Where not shown, error bars are within the symbol. The optimum stimulation of [^3^H]ryanodine binding was significantly greater for ATP than for PPPi (**p < 0.01 (0.0065)).

Not all channels responded to PPPi in the same manner. In 8 out of 15 experiments (53%), PPPi produced irreversible inactivation at high doses and this type of response can be observed in Fig. 3A. The high dose of PPPi (10 mM) closed the channel, with only occasional very brief openings being observed. Channels shut down completely within 150 s (range 56–143 s) and, as shown in the bottom trace, the inactivation could not be reversed by perfusing away the PPPi (n = 8). At concentrations of PPPi <10 mM we did not observe channel inactivation. This does not mean that inactivation would not occur at lower concentrations. It is likely that inactivation would be concentration and time dependent but it is not practical to investigate time-dependent effects of ligands due to the short lifetime of a bilayer.

### [^3^H]ryanodine binding studies

Since PPPi activates some channels and inactivates others it is useful to understand how PPPi activates a population of RyR2 channels and how effective it is in comparison to ATP. [^3^H]ryanodine binding experiments can be utilised to investigate these questions allowing us to examine populations of channels in their native membranes. Figure 3B demonstrates that PPPi was far less effective than ATP at stimulating the binding of [^3^H]ryanodine to cardiac SR vesicles, exhibiting a lower maximum level of binding and higher EC_50_.

**Figure 4:**
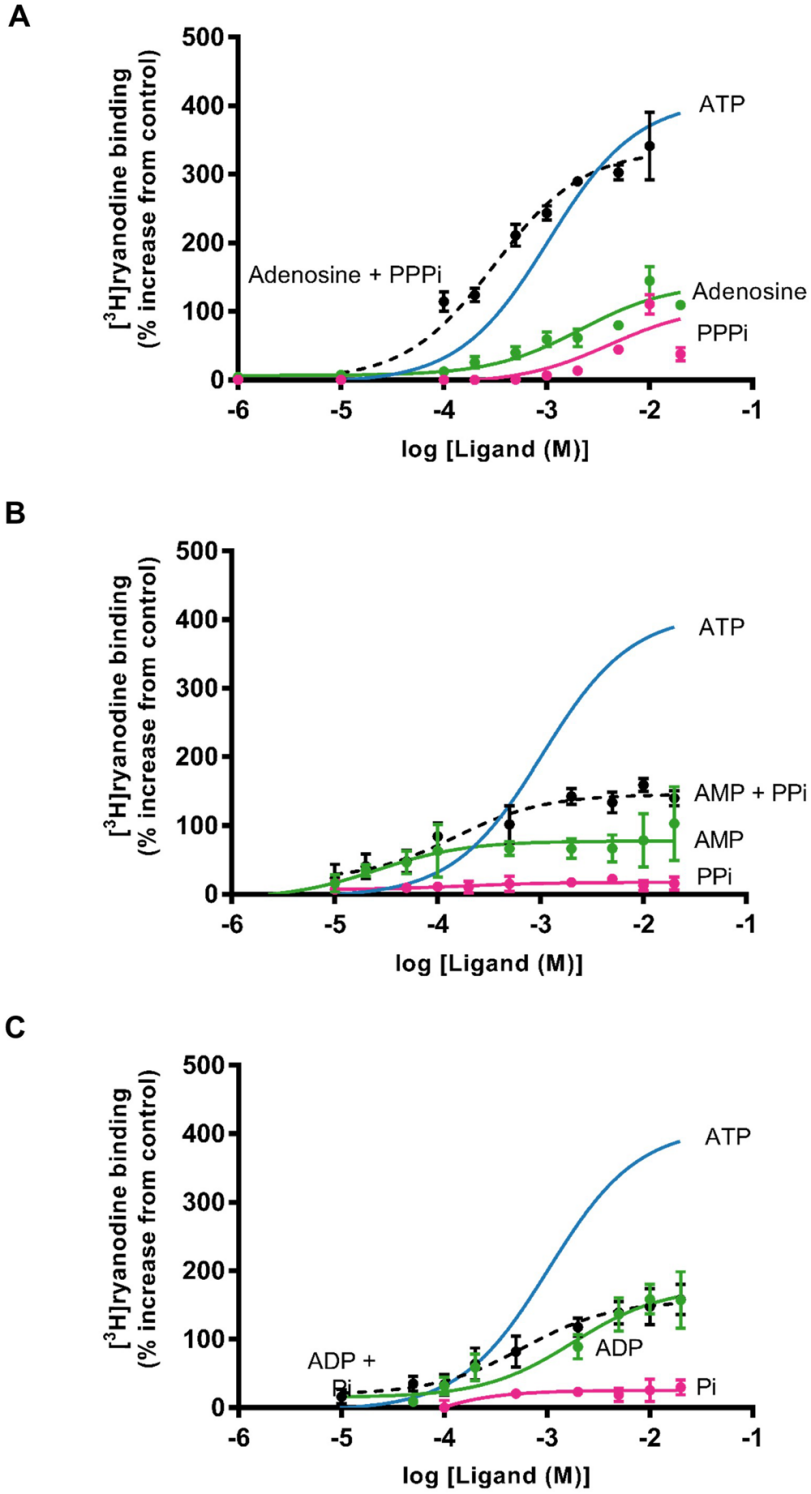
Investigating the ability of ATP fragments to stimulate [^3^H]ryanodine binding as effectively as ATP alone. For clarity, in each graph, the ATP data from Fig. 3B is shown as a solid blue line. (**A**) Comparison of the effects of PPPi alone (pink), adenosine alone (green) and adenosine in the presence of 10 mM PPPi (black dashed line). (**B**) Comparison of the effects of PPi alone (pink), AMP alone (green) and AMP in the presence of 10 mM PPi (black dashed line). (**C**) Comparison of the effects of Pi alone (pink), ADP alone (green) and ADP in the presence of 10 mM Pi (black dashed line). Where combinations of ligands were used, they were added simultaneously at the start of the incubation period. The addition of Pi to ADP and PPi to AMP did not significantly increase binding above that observed with ADP (p = 0.2101) or AMP (p = 0.1917) alone whereas the addition of PPPi significantly increased binding above that observed with adenosine alone (**p < 0.01 (0.0065)). The error bars indicate SEM (n = 5). Where not shown, error bars are within the symbol.

Since PPPi alone can activate RyR2 but less effectively than ATP, we considered whether different fragments that make up the ATP molecule can, when added together, promote the same changes in channel gating as ATP. Figure 4 shows three experiments where we investigated whether Pi + ADP, PPi + AMP and PPPi + adenosine could substitute for ATP. For comparison, the dose-response relationship for ATP (from Fig. 3B) is shown as a blue line. Independently, ADP, AMP and adenosine are unable to stimulate [^3^H]ryanodine binding to the same level as ATP. Adding the missing phosphate component simultaneously with ADP or AMP also does not reproduce the effects of ATP. The maximum level of [^3^H]ryanodine binding observed with ADP is indistinguishable from that obtained in the presence of Pi + ADP. With AMP, the simultaneous presence of AMP + PPi appears to produce approximately equivalent effects to those expected by adding the sum of the individual actions of AMP and PPi. With adenosine and PPPi, however, the simultaneous presence of both ligands causes a greater stimulation of [^3^H]ryanodine binding than the sum of their individual effects although the presence of both ligands does not exactly reproduce the effects of ATP.

**Figure 5:**
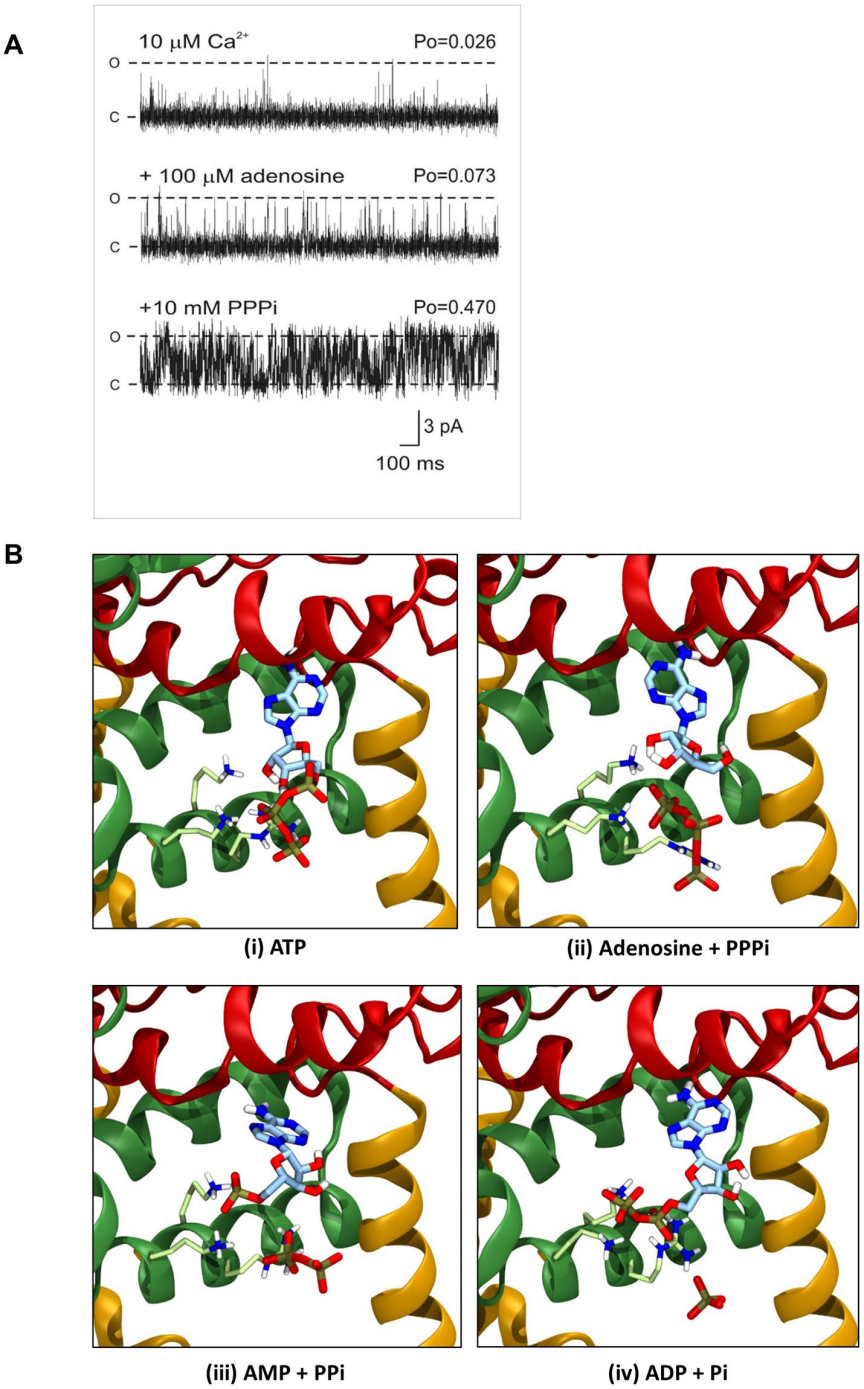
Effects of adenosine plus PPPi on RyR2 gating and the docking of complementary fragments of ATP. (**A**) Current fluctuations through a typical single RyR2 channel recording in the presence of 10 μM Ca^2+^ (top trace), after addition of 100 μM cytosolic adenosine (middle trace) and the subsequent addition of 10 mM cytosolic PPPi in the continued presence of 100 µM adenosine (bottom trace). The holding potential was 0 mV. O and C represent the open and closed channel levels, respectively. (**B**) The RyR2 ATP binding site with the most ATP-like docking results for (i) ATP, (ii) adenosine and PPPi, (iii) AMP and PPi, and (iv) ADP and Pi. In each case, the nucleoside ligand was docked first, the most ATP-like pose was adopted as part of the protein, and the phosphate ligand subsequently docked into the same site.

It is possible that adenosine and PPPi can simultaneously slot into the ATP binding site and that the presence of adenosine prevents the PPPi-induced inactivation that occurs in more than 50% of the single-channel experiments. We investigated this at the single-channel level. Figure 5A illustrates that, as previously described^29^, adenosine alone causes only a small increase in Po; an effect brought about by an increase in the frequency of channel opening. Subsequent addition of 10 mM PPPi caused a large increase in Po but channel inactivation was not observed in this experiment or in five similar experiments using 100 μM adenosine. Table 1 shows the Po values in the presence of 100 μM, 1 mM and 10 mM adenosine, before and after addition of 10 mM PPPi. Inactivation was not observed in any experiment (n = 16) demonstrating that adenosine does protect against this action of PPPi.

**Table 1:** Effect of adenosine on the ability of PPPi to activate RyR2.

Adenosine concentration	Mean RyR2 open probability (Po) ± SEM	
	Adenosine alone	Adenosine + 10 mM PPPi
100 μM	0.068 ± 0.014 (n = 6)	0.374 ± 0.056 (n = 6)**
1 mM	0.080 ± 0.032 (n = 5)	0.331 ± 0.099 (n = 5)
10 mM	0.155 ± 0.081 (n = 5)	0.572 ± 0.107 (n = 5)**

To avoid inaccuracies in the [adenosine] due to the limits of adenosine solubility, 1 and 10 mM adenosine were perfused into the cytosolic chamber following fusions of the SR vesicles and therefore it is not possible to determine a ‘control’ value in the absence of adenosine (although comparison with other channels at 10 µM cytosolic Ca^2+^ could be made). 100 μM adenosine was obtained by addition of a 10 mM stock solution of adenosine, and in this case the control Po in the presence of 10 μM Ca^2+^ alone was 0.038 ± 0.009 (SEM, n = 6). Inactivation was not observed in any experiment but since adenosine binds reversibly to RyR2, one might expect to see occasional channel inactivation that occurred less frequently as the concentration of adenosine increased. However, this is difficult to show at the single-channel level and hence the [^3^H]ryanodine binding data in Fig. 4A is likely to provide a more complete picture of overall RyR2 activities for populations of channel. Note that adenosine is a partial agonist at the RyR2 ATP site^29^. Adenosine alone does not increase Po above approx. 0.15, thus in the presence of an agonist with higher efficacy such as ATP or PPPi, it can appear to act as an antagonist. We have previously demonstrated that 100 μM and 1 mM adenosine inhibit channel activation by 1 mM ATP by 57% and 74% respectively^29^. Therefore, unless PPPi and adenosine are both simultaneously aligned together in the ATP binding site (and the probability of this occurring will increase with increasing concentrations of adenosine), adenosine will appear to act as an antagonist. This is in line with the results since 10 mM adenosine and 10 mM PPPi gives the highest Po. (Mean values ± SEM are shown. **p < 0.01. p values relative to adenosine alone).

### Docking

To examine whether adenosine and PPPi, as well as other combinations, could occupy the ATP site at the same time, we conducted a molecular modelling study to investigate a structural explanation for the varied gating effects of ATP-like ligands. No RyR2-ATP bound structure has yet been reported, and therefore we compared the cryo-EM structure of RyR2 in the open state (PDB ID 5GOA)^16^ with that of the structure of RyR1 reported by des Georges *et al*.^17^ in the presence of ATP (PDB ID 5TAS). Consistent with the high level of sequence identity between the two proteins, we found that the ATP site is largely conserved with an RMSD of less than 2 Å for all C_α_ atoms of residues within 10 Å of ATP. Figure 1A (inset) shows an overlay between the RyR1 ATP site and the equivalent region of the RyR2 structure, illustrating the similarity. The protein and ligands were prepared for docking as described in the methods section.

We first attempted to verify that a docking approach studying ATP binding to RyR2 could reproduce the observed binding mode of ATP seen in the cryo-EM structure of ATP bound RyR1 by des Georges *et al*.^17^. Initial attempts were unsuccessful and appeared to produce a variety of binding modes, none of them with the adenine base placed in the RyR2 hydrophobic pocket. Close inspection of the RyR2 site revealed the M^4885^ residue appeared to be obstructing the hydrophobic pocket of the site. An alternate rotamer, more like that observed in RyR1, was selected after a test run with a flexible methionine side chain gave better results when M^4885^ was rotated away from the crystal orientation. Further attempts to dock ATP revealed binding modes which were similar to the ATP binding mode experimentally observed in RyR1^17^, with the adenine core buried in the hydrophobic pocket and phosphate groups positioned to be stabilised by interactions with the positive K^4167^, K^4170^ and R^4171^ lining the pocket, illustrated in Fig. 1B. It also appeared that the N1 adjacent to the amino group of the adenine nucleotide (Fig. 2A) is positioned to hydrogen bond with the C^4889^ residue (bond angle 124.5° and length 2.5 Å) (inset to Fig. 1B). In the case of RyR1, Yan *et al*.^30^ noted the presence of a zinc-finger motif in the C-terminal subdomain, which is adjacent to the adenine binding pocket^30,31^. The Zn^2+^ site is also present in RyR2 and we therefore compared the results for docking of the ATP ligand into a protein structure with or without Zn^2+^ bound (Table 2). The binding energies with Zn^2+^ present were slightly lower than without, but as the poses generated without Zn^2+^ looked much more similar to the cryo-EM RyR1 structure (example shown in Fig. 1), the Zn^2+^ ion was removed for further docking calculations.

**Table 2:** Docking scores of ATP-like ligands.

Ligand	Mean binding energy of most ATP-like cluster (kcal/mol)	No. of clusters (different binding modes among the 50 generated poses)	No. of results in the most ATP-like cluster/binding mode
ATP	−2.60	38	4
ATP (+Zn)	−3.25^#^	33	6^#^
PPPi (deep in site)	−3.55	11	4
PPPi (on edge of site)	−2.95*	11	22*
Adenosine	−1.07	28	3
AMP	−0.85	31	2
ADP	−0.73	32	2
AMP-PCP	−1.73	33	2
GTP	+3.63	34	2
**Phosphate docking with nucleoside already bound**			
PPPi (+adenosine)	−5.34	11	24
PPi (+AMP)	−3.39	5	31
Pi (+ADP)	−2.90	8	20

Shown are the mean binding energies (kcal/mol) of the most ATP-like cluster of results predicted by Autodock for the various ligands listed (column 2), the total number of clusters (i.e binding modes grouped together by similarity, column 3), and the number of poses that were in the most ATP-like cluster (column 4). The table provides an indication of the extent to which the results predicted by Autodock were similar to the RyR1 ATP-bound des Georges *et al*. (2016) structure.

*PPPi has two different interesting binding modes. The first-mentioned is the lowest-energy one which is fairly deep in the adenine pocket. In the second-mentioned cluster, the triphosphate occupies a position in which it would overlap with the triphosphate of ATP.

^#^For ATP (with Zn^2+^ bound to the protein) none of the poses obtained were ‘ATP-like’ when compared with the des Georges *et al*. (2016) RyR1 ATP-bound RyR1 structure. In this case we therefore report the energy of the lowest energy cluster.

We then used the same docking parameters to dock ADP, AMP and adenosine into the putative RyR2 ATP site. Our objectives were two-fold: firstly to investigate if ATP derivatives bind in a similar way to ATP, and, if so, to focus the analysis on those poses that bind in a similar way to that seen by des Georges *et al*. (2016). For all docked ATP derivatives, poses with the base in the hydrophobic pocket and the phosphates, if present, interacting with the positive residues were observed. The representative pose was chosen as the pose with the lowest predicted binding energy of the poses that bound in an ATP-like manner, and these representative ‘ATP-like’ binding modes are shown in Fig. 5B. The binding energies predicted by Autodock are shown in Table 2. Our second aim was to use the most ‘ATP-like’ pose of each ATP derivative for a second round of docking calculations. These binding modes could be fixed as part of the protein to explore the possibility of then subsequently docking various phosphate groups simultaneously with the ATP derivatives.

**Figure 6:**
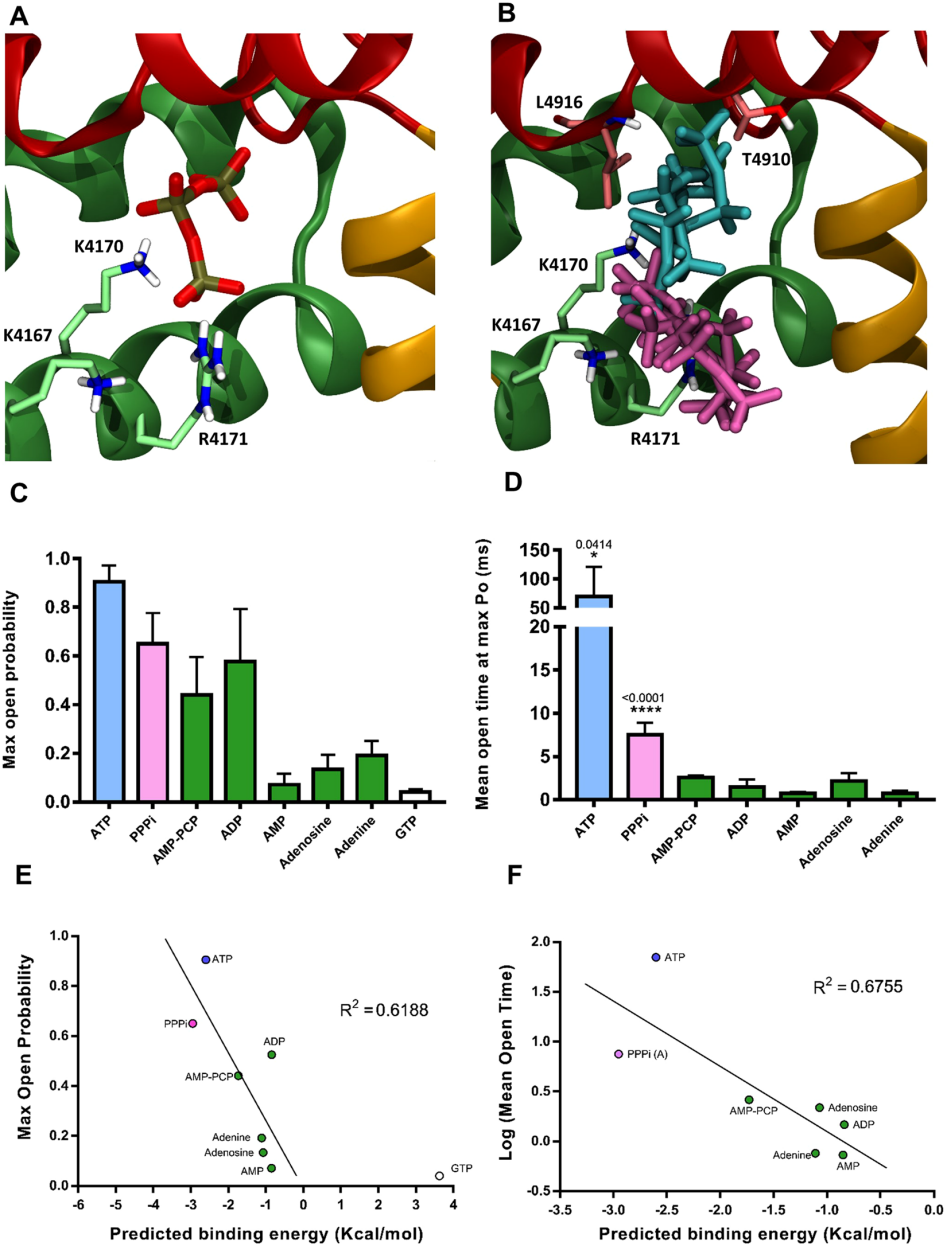
PPPi docking into the RyR2 ATP site and summary of the effects of ATP analogues on RyR2 gating. (**A**) A representative pose from the lowest energy cluster for docking of PPPi into the RyR2 ATP site. The residues R4171, K4167 and K4170 are indicated. (**B**) Poses from two clusters for docking PPPi into the RyR2 ATP site. Four poses from each cluster are shown, the ATP-like cluster is shown in pink, while the cluster binding deeper in the pocket (with lower energy) is shown in cyan. Residues that appear to interact with these poses are also indicated. (**C**) Comparison of the ability of ATP and other adenine or phosphate containing molecules to increase Po. The maximum Po obtained in single-channel experiments (with 10 μM free Ca^2+^) by the compounds indicated in the bar chart is shown. (**D**) The mean open time at maximum Po for ATP and various fragments or analogues of ATP is shown. Only ATP and PPPi significantly increased mean open time relative to control (for ATP p = 0.0414 and for PPPi p < 0.0001). The data includes results from this study and from our previous work on adenine nucleotides performed under identical experimental conditions only^3,6,10,39^. The error bars are SEM (n ≥ 4). Correlation between the maximum Po (**E**) or the mean open time (**F**) obtained for each ligand and Autodock predicted mean binding energies (kcal/mol) for the various ligands listed. As GTP did not bind in the ATP site, the point is shown, but the value has not been included in the statistical analysis. The Pearson correlation coefficients (R^2^) were 0.6188 and 0.6755, and the p values were 0.0359 and 0.023, respectively, thus showing significant correlation (p < 0.05).

We also investigated the favoured binding pose of PPPi when docked alone, which is shown in Fig. 6A. When PPPi was docked alone, and therefore not connected to an adenine nucleotide, the binding poses were more varied than when the triphosphate group is incorporated into an ATP molecule. The lowest energy binding mode (cluster) appeared to be bound deeper in the hydrophobic pocket, within 2 Å of the L^4916^ and T^4910^ residues, in a favourable position for hydrogen bonding (Fig. 6A,B) while still also able to interact with some of the positive residues. The other dominant binding mode contained poses clustered around the lysine and arginine residues lining the site, and similar to that of the triphosphate group of a full ATP molecule (Fig. 6B). Docking ATP fragments into the putative RyR2-ATP binding site suggests that the ATP breakdown compounds, ADP, AMP, and adenosine, can all bind into this site in a similar manner to ATP (Fig. 5B) as can the non-hydrolysable analogue of ATP, AMP-PCP (Table 2). When comparing the Autodock-predicted mean binding energies for the clusters which bind similarly to ATP (Table 2), it is clear that ATP and PPPi have the lowest predicted binding energies. AMP-PCP, ADP, adenosine and AMP appear to bind less well, all with slightly higher predicted binding energies, while GTP has a much higher predicted binding energy suggesting that it does not bind very well in the ATP site. The accuracy of predicted binding energies from docking calculations using a general scoring function is usually low^32^, however, Autodock is able to produce ATP-like binding modes for each of these ligands and there appears to be a broad relationship between the Autodock-predicted mean binding energy and the ability of the ligand to induce high Po and long open states (Fig. 6E,F). This gives us confidence that our model is useful for understanding ligand binding in the RyR2 ATP site, keeping in mind that the resolution of the structure is relatively poor at 4.2 Å.

To investigate the effect of adding multiple ligands, Pi, PPi and PPPi were docked into structures already binding ADP, AMP or adenosine, respectively. Figure 5B compares the lowest energy ‘ATP-like’ pose of the full ATP molecule with those obtained by the docking of the various adenine nucleotides with subsequent docking of their complementary phosphate groups. We observed that the presence of ADP in the binding site appeared to obstruct Pi from closely interacting with the positive residues lining the pocket. A similar situation was seen in the case AMP and PPi. However, when adenosine was bound in the pocket, PPPi could make a close approach to the positive residues and therefore occupy a similar position to that seen with ATP.

## Discussion

ATP has long been recognised as a crucial physiological regulator of RyR2 and although many adenine-based ligands can activate RyR2 with varying effectiveness, we have shown previously^10^ that the negative charge of the phosphate groups is the driver for activating the channels to high Po. In this study, we now demonstrate that the triphosphate group alone is capable of activating RyR2 to high levels. By constructing a model of the RyR2 ATP binding site, based on the recent cryo-EM structures of the ATP site within RyR1^17^, we have been able to make key predictions about the binding of ATP, PPPi and other fragments of ATP that can explain their functional behaviour.

The sequence identity between RyR1 and RyR2 is approximately 65% across their full sequence^33^ however, for the 50 residues within 10 Å of the bound ATP molecule, only three amino acids differ. Comparison of the cryo-EM structures of RyR1 with ATP bound and RyR2 without ATP bound confirm that the ATP site is highly conserved between the two isoforms with an RMSD of less than 2 Å for the before-mentioned 50 residues. This is not unexpected given the similarity of the effects of ATP on the gating of RyR1 and RyR2 isoforms^2,3^. Hence, in the absence of a RyR2-ATP bound structure, it is most likely that the ATP site is common between RyR1 and RyR2. Modelling the ATP binding site based on the des Georges *et al*.^17^ RyR1-ATP bound structure is therefore expected to yield the most reliable approximation of adenine nucleotide interactions with RyR2 that is currently possible.

It is interesting that, alone, PPPi causes marked activation in approximately 50% of single channel experiments in contrast to Pi and PPi which produce only slight increments in RyR2 Po or [^3^H]ryanodine binding. With 10 μM cytosolic Ca^2+^, the maximum Po achievable with PPPi is approximately 0.6, hence in order of ability to activate RyR2 to high levels, PPPi positions into the nucleotide series as follows: ATP > PPPi > ADP ≥ AMP-PCP > adenine ≥ adenosine ≥ AMP > GTP^10,29^ (Fig. 6C). Thus, after ATP, PPPi is the next most effective ligand to cause high Po. The mechanism by which Po is increased is important and Fig. 6D shows that ATP can lengthen the mean open time from approximately 1 ms (in control conditions of 10 µM Ca^2+^) to 70 ms and because of this property can almost fully open the channel. PPPi is the only other ligand that can increase mean open time above 5 ms and even this effect is rather minor in comparison to that of ATP.

The ability of PPPi to increase Po and prolong open states was unexpected. Since GTP (Fig. 6C) and other non-adenine nucleotides do not significantly activate RyR channels^6,8,34^ and since Pi has minimal effects on RyR2^3,9^, it seemed that the adenine base was required for binding to the RyR ATP site. Although we show that the adenine base is not essential, it is still important. Not only does PPPi have a lower ability to increase Po in comparison with ATP but the affinity of PPPi for RyR2 is lower (PPPi EC_50_ for single-channel Po = 2.4 mM; ATP EC_50_ = 220 μM)^3^. Additionally, PPPi possesses novel inactivating properties (approximately 50% of channels are shut down by PPPi). The single-channel data shown in Fig. 5A and listed in Table 1 demonstrate that adenosine can protect against the PPPi-induced inactivation but also shows that the intact ATP molecule is required for full efficacy. Under identical experimental conditions, the maximum Po that ATP can induce is approximately 0.9 whereas adenosine + PPPi together activate to a level within the range 0.3–0.6. As the triphosphate moiety is essential for the full agonist properties of the ATP molecule, it suggests that a strong interaction with the positively charged lysine and arginine residues, or perhaps sufficient shielding of the positive charge, is necessary for driving channel opening and that this cannot be achieved with adenine nucleosides with fewer phosphates (ADP, AMP). Hence, while the adenine moiety is not an absolute requirement, it is a must for tight binding within the ATP-binding pocket and optimal positioning to enable the phosphates to cause long open states and high Po. This is also consistent with our observation from the modelling data, that the C^4889^ residue appears important for binding of the adenine core. The inset of Fig. 1B shows the adenine ring of ATP is ideally positioned for a hydrogen bond between the N1 atom adjacent to the NH_2_ group and the polar Hγ atom of the C^4889^ residue. A similar interaction would not be possible with a guanosine nucleotide in the same position, as in this case the N1 is already bound to a hydrogen atom and hence is not available as a hydrogen bond acceptor. Consistent with this we found that when GTP was docked in the RyR2 ATP site under the same conditions, none of the poses resembled ATP and all poses had unfavourable positive predicted binding energies (Table 2). Although we cannot say, based on the current data, that this interaction with C^4889^ is sufficient to discriminate between GTP and ATP, our modelling results suggest that an adenine nucleotide is required for full channel activation.

The [^3^H]ryanodine binding data together with our predictions from molecular modelling of ATP fragments docking into the RyR2 ATP site suggest possible underlying mechanisms for the actions of PPPi. Our docking results suggest that when PPPi is alone and not connected to an adenine nucleotide, it preferentially binds in one of two locations within the ATP binding pocket and that one favourable location is surprisingly deep within the pocket, reducing its potential for interacting with the positively charged residues which line the pocket (compare Figs 5B with 6A and B). Irreversible binding of PPPi in this location may then lead to channel closure. Our functional data suggests that this is possible. In single channel experiments, pre-addition of adenosine protects against irreversible channel closure by PPPi alone. Of course, we cannot exclude the possibility that PPPi binds to other sites on the RyR2 protein. However, the observation that in the presence of adenosine, channel opening is promoted, suggests that adenosine directs the PPPi molecule into a favourable binding site for causing channel opening, possibly by filling the site where the adenine portion of ATP tends to dock such that PPPi is forced to dock nearer the preferred location for the triphosphate group of ATP. PPPi is also more effective at stimulating [^3^H]ryanodine binding in the presence of adenosine and one explanation for this is that when both adenosine and PPPi dock into the ATP binding site, PPPi no longer irreversibly closes 50% of the channels and, instead, increases Po more effectively than either compound alone. Again, our docking results support this hypothesis and suggest that the preferred binding pose for PPPi, when adenosine is also bound, is more similar to that for the triphosphate groups of the ATP molecule. We therefore suggest that the adenine ring of ATP provides a directing effect, encouraging productive binding of PPPi in a manner which leads to activation, and prevents binding in a mode which leads to inactivation.

The context in which the components of the ATP molecule bind to RyR2 is important: combinations of fragments of the ATP molecule (ADP + Pi, AMP + PPi or adenosine + PPPi) cannot exactly reproduce the effects of ATP. Even for adenosine and PPPi, the combined effect of these two fragments causes a slightly lower maximum Po level and a lower stimulation of [^3^H]ryanodine binding than would be obtained with ATP alone. The docking experiments highlighted in Fig. 5 and Table 2 provides a potential explanation. In the case of ADP, the adenine core of the molecule is predicted to bind in the hydrophobic pocket in a similar manner to ATP, with the diphosphate tail coordinating to both lysine residues (illustrated in Fig. 5B), hence, the reduced interaction of ADP with the arginine residue may explain its reduced efficacy. When the remaining phosphate now is docked in addition, it is not possible for Pi to coordinate to any of these residues, as to do so would create unfavourable repulsion between the Pi and phosphates of ADP. This explains why the addition of Pi has no effect (Fig. 4C). For AMP, the same is true. However, with only one phosphate, the AMP can only coordinate to one lysine, leaving the other available for interaction with PPi. We therefore see that the added PPi can interact with both a lysine and an arginine residue, and so a small increase in channel activity is observed. Finally, adenosine alone, with no phosphate groups, is no longer able to interact with the positive residues. These residues are now much more available for interaction with the added PPPi ligand. As adenosine is not charged, the lack of repulsion allows PPPi and adenosine together to bind in a similar manner to an ATP molecule (Fig. 5B).

The knowledge of how the fragments of the ATP molecule affect RyR2 function provides important physiological and pathophysiological insight. In healthy cardiac cells, ATP concentrations are expected to be within the range 4–12 mM^35^ whereas the levels of many other adenine nucleotides are very low or barely detectable^7,36^. It is therefore expected that ATP would normally occupy the adenine nucleotide binding sites providing a consistent activation of RyR2 that is dependent on the cytosolic free [Ca^2+^]. The adenosine portion of the ATP molecule prevents the triphosphate moiety from creating an irreversibly closed mode, which would otherwise cause major disruption of SR Ca^2+^-release. Myocardial ischaemia is associated with rises in the levels of Pi, ADP, AMP and adenosine and eventually with decreases in ATP levels; the changes depending on the duration of the ischaemic insult^7,36^. It is clear from our experiments that multiple components of ATP would be unable to mimic the intact ATP molecule and that activation of RyR2 would be reduced, limiting the amount of Ca^2+^ released from the SR. The extent of disruption to EC-coupling would depend on the duration of ischaemia and the exact levels of ATP and its breakdown products.

From a pharmacological point of view, the ATP binding site on RyR channels could also be responsible for the off-target effects of a range of other drugs as many different types of adenine-based compounds can bind here. For example, there are a growing number of nucleoside analogue drugs such as vidarabine (antiviral) and immucillin-A (ebola) which are structurally related to ATP^37^. Further understanding of the structural features of ATP analogues that result in RyR2 activation or inhibition will therefore facilitate drug discovery by early identification of undesirable chemical motifs in drug candidates.

In summary, we provide the first structure-based investigation into the binding of ligands in the RyR-ATP binding site, affording new insight into the likely mechanisms by which ATP and analogues regulate RyR2 gating. In future studies it is hoped that dynamical simulations can be performed. These will be challenging given the huge size of RyR and the complex synergistic effects of the various RyR regulators but have the potential for greater mechanistic understanding of ligand-driven RyR gating changes. Such knowledge is likely to have practical significance for improving treatments in cardiac disease where abnormalities in SR Ca^2+^ release contribute to impaired contractile function and the generation of life-threatening arrhythmias.
